# Effect of Written Exposure Therapy vs Cognitive Processing Therapy on Increasing Treatment Efficiency Among Military Service Members With Posttraumatic Stress Disorder: A Randomized Noninferiority Trial

**DOI:** 10.1001/jamanetworkopen.2021.40911

**Published:** 2022-01-11

**Authors:** Denise M. Sloan, Brian P. Marx, Patricia A. Resick, Stacey Young-McCaughan, Katherine A. Dondanville, Casey L. Straud, Jim Mintz, Brett T. Litz, Alan L. Peterson

**Affiliations:** 1 Behavioral Science Division, National Center for PTSD at VA Boston Healthcare System, Boston, Massachusetts; 2 Department of Psychiatry, Boston University School of Medicine, Boston, Massachusetts; 3 Department of Psychiatry and Behavioral Sciences, Duke Health, Durham, North Carolina; 4 Department of Psychiatry and Behavioral Sciences, The University of Texas Health Science Center at San Antonio; 5 Research and Development Service, South Texas Veterans Health Care System, San Antonio; 6 Department of Psychology, The University of Texas at San Antonio; 7 Massachusetts Veterans Epidemiological Research and Information Center, VA Boston Healthcare System, Boston; 8 Department of Psychological and Brain Sciences, Boston University, Boston, Massachusetts

## Abstract

**Importance:**

Posttraumatic stress disorder (PTSD) occurs more commonly among military service members than among civilians; however, despite the availability of several evidence-based treatments, there is a need for more efficient evidence-based PTSD treatments to better address the needs of service members. Written exposure therapy is a brief PTSD intervention that consists of 5 sessions with no between-session assignments, has demonstrated efficacy, and is associated with low treatment dropout rates, but prior randomized clinical trials of this intervention have focused on civilian populations.

**Objective:**

To investigate whether the brief intervention, written exposure therapy, is noninferior in the treatment of PTSD vs the more time-intensive cognitive processing therapy among service members diagnosed with PTSD.

**Design, Setting, and Participants:**

The study used a randomized, noninferiority design with a 1:1 randomization allocation. Recruitment for the study took place from August 2016 through October 2020. Participants were active-duty military service members diagnosed with posttraumatic stress disorder. The study was conducted in an outpatient setting for service members seeking PTSD treatment at military bases in San Antonio or Killeen, Texas.

**Interventions:**

Participants received either written exposure therapy, which consisted of 5 weekly sessions, or cognitive processing therapy, which consisted of 12 twice-weekly sessions.

**Main Outcomes and Measures:**

Participants were assessed at baseline and at 10, 20, and 30 weeks after the first treatment session. The primary outcome measure was PTSD symptom severity assessed with the Clinician-Administered PTSD Scale for *DSM-5 *(CAPS-5). Noninferiority was defined as the difference between the 2 groups being less than the upper bound of the 1-sided 95% CI–specified margin of 10 points on the CAPS-5.

**Results:**

Overall, 169 participants were included in the study. Participants were predominantly male (136 [80.5%]), serving in the Army (167 [98.8%]), with a mean (SD) age of 34 (8) years. Eighty-five participants were randomly assigned to written exposure therapy, with 65 (76.5%) completing all treatment sessions, and 84 to cognitive processing therapy, with 47 (54.8%) completing all treatment sessions. Findings indicated that written exposure therapy was noninferior to cognitive processing therapy, with the largest difference in change in outcome between the treatment conditions of 3.96 points on the outcome measure. The 1-sided 95% CI upper limit was less than 10 points across time points in both groups and ranged from 4.59 at week 30 to 6.81 at week 10. Within-condition effect sizes ranged from a Cohen *d* of 0.48 for the written exposure therapy group in the intention-to-treat analysis at week 10 to 0.95 for the cognitive processing therapy group in the per-protocol analysis at week 10, and between-condition effect size ranged from 0.06 in the intention-to-treat analysis at week 30 to 0.22 in the per-protocol analysis at week 10.

**Conclusions and Relevance:**

In this randomized clinical trial, support was found for an effective and more efficient PTSD treatment approach for service members. Future research should determine who does and does not benefit from PTSD treatment to best maximize treatment outcomes.

**Trial Registration:**

ClinicalTrials.gov Identifier: NCT03033602

## Key Points

**Question:**

Is a 5-session, trauma-focused written exposure therapy treatment noninferior to a more time-intensive, trauma-focused cognitive processing therapy treatment for active-duty service members diagnosed with posttraumatic stress disorder (PTSD)?

**Findings:**

In this randomized noninferiority clinical trial that included 169 men and women activity-duty service members, written exposure therapy was found to be noninferior to cognitive processing therapy. Dropout rates for written exposure therapy were significantly lower than for cognitive processing therapy.

**Meaning:**

These findings suggest that written exposure therapy, a more efficient treatment approach for PTSD than cognitive processing therapy, should be considered for military service members.

## Teaser

This randomized noninferiority trial investigates whether a 5-session written exposure therapy is noninferior in the treatment of posttraumatic stress disorder (PTSD) vs 10-session cognitive processing therapy among service members diagnosed with PTSD.

## Introduction

Posttraumatic stress disorder (PTSD) is a prevalent and debilitating disorder. The prevalence of PTSD is greater among military service members^1^ than among the general population.^2^ Prior studies suggest that service members with PTSD may be treated effectively with evidence-based treatments, such as prolonged exposure^3^ and cognitive processing therapy (CPT).^4,5^ Unfortunately, service members are often reluctant to seek mental health care,^6^ and when they do receive care for PTSD, a higher percentage drop out of treatment prematurely compared with civilians who receive PTSD treatment.^7^ Although there are numerous reasons for not seeking PTSD treatment or dropping out prematurely, one possible explanation is that those with PTSD have competing demands on their time.^8^ Evidence-based PTSD treatments typically require 8 to 15 sessions, as well as between-session assignments that can require a substantial amount of time to complete.^9,10^ The time investment may be challenging for patients. Many service members struggling with PTSD may not have an opportunity to engage in these treatments, especially if they are undergoing intensive military training assignments, are required to travel on temporary duty assignments, or are preparing for a deployment.

An effective yet brief PTSD treatment may increase the likelihood that service members seek and complete treatment. Written exposure therapy^11^ (WET) is a 5-session PTSD treatment with no between-session assignments that is efficacious in the treatment of civilians^12^ as well as noninferior to CPT in a mixed trauma sample.^13^

This study examined whether WET is noninferior to the CPT protocol that does not include written trauma accounts (and which may be associated with faster treatment gains^14^) in PTSD symptom change and whether it reduces treatment dropout among service members. In addition, among studies treating service members, CPT without written accounts has been investigated more frequently than the full protocol version.^4,5,15^ Based on prior findings of WET, we expected WET would be noninferior to CPT at all assessment periods included in the study and associated with better treatment retention than CPT.

## Methods

The study followed the Consolidated Standards of Reporting Trials (CONSORT) reporting guideline for trial studies.^16^ The study was approved by the institutional review boards at the University of Texas Health Science Center at San Antonio, Duke University, and VA Boston Healthcare System. The US Army Medical Research and Development Command Human Research Protection Office monitored all regulatory approvals.

### Participants

Participants were 169 active-duty US military personnel stationed at military bases in San Antonio or Killeen, Texas, who were seeking treatment for PTSD. Table 1 presents the demographic characteristics of the sample. Of the 169 participants randomized, 84 were randomized to CPT and 85 were randomized to WET.

**Table 1.** Baseline Demographics per Treatment Condition and for the Total Sample

Characteristic	Participants, No. (%)^a^		
	CPT (n = 84)	WET (n = 85)	Total (n = 169)
Gender			
Male	67 (79.8)	69 (81.2)	136 (80.5)
Female	17 (20.2)	16 (18.8)	33 (19.5)
Age, mean (SD), y	33.32 (8.22)	33.98 (8.68)	33.65 (8.43)
Race			
African American	29 (34.5)	28 (32.9)	57 (33.7)
White	33 (39.3)	26 (30.6)	59 (34.9)
Hispanic	17 (20.2)	25 (29.4)	42 (24.9)
Other^b^	5 (6.0)	6 (7.1)	11 (6.5)
Marital status			
Married	67 (79.8)	63 (74.1)	130 (76.9)
Single/never married	6 (7.1)	10 (11.8)	16 (9.5)
Divorced/separated	11 (13.1)	12 (14.1)	23 (13.6)
Education			
HS degree or equivalent	22 (26.2)	18 (21.2)	40 (23.7)
Some college	45 (53.6)	59 (69.4)	104 (61.5)
4-y degree or higher	17 (20.2)	8 (9.4)	25 (14.8)
Army	84 (100)	83 (97.6)	167 (98.8)
Enlisted rank	75 (89.3)	79 (92.9)	154 (91.1)
Duty, No./total No. (%)			
Combat Arms	27/66 (40.9)	22/69 (31.9)	49/135 (36.3)
Combat Support	15/66 (22.7)	14/69 (20.3)	29/135 (21.5)
Combat Service Support	24/66 (36.3)	33/69 (47.8)	57/135 (42.2)
Months in military, mean (SD)	152.82 (92.38)	157.76 (87.73)	155.31 (89.84)
No. of deployments, No./total No. (%)			
0	0	1/70 (1.4)	1/137 (0.7)
1	15/67 (22.4)	13/70 (18.6)	28/137 (20.4)
2	12/67 (17.9)	24/70 (28.2)	36/137 (26.3)
3	21/67 (31.4)	17/70 (34.3)	38/137 (27.7)
4	19/67 (28.4)	15/70 (21.4)	34/137 (24.8)

Abbreviations: CPT, cognitive processing therapy; HS, high school; WET, written exposure therapy.

^a^ Counts vary based on available data.

^b^ Included individuals who identified as American Indian or Alaska Native, Asian, Pacific Islander, or biracial.

To be included in the study, individuals had to be aged at least 18 years, meet diagnostic criteria for PTSD, and plan to remain in the geographic area for at least 3 months. Participants taking psychotropic medication had to agree to remain on a stable dose for at least 4 weeks prior to study entry. Exclusion criteria included current suicide or homicide risk meriting crisis intervention, active psychosis or mania, and moderate to severe traumatic brain injury (TBI). The Figure presents for specific information on recruitment and participation.

**Figure.**
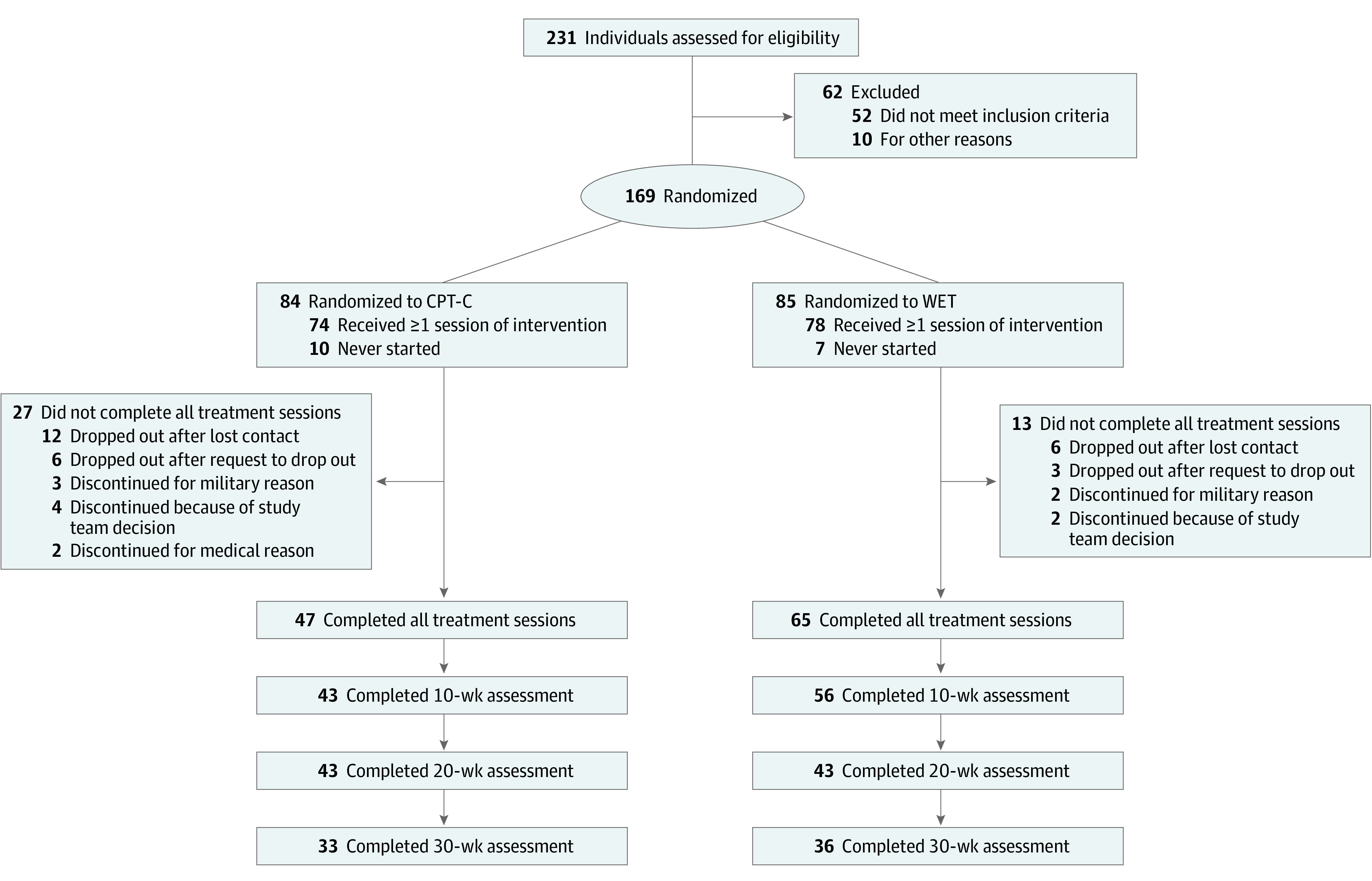
Study Flow Diagram CPT-C indicates cognitive processing therapy–cognitive therapy only; WET, written exposure therapy.

Recruitment methods included advertisements and direct referrals from military health care practitioners. Between August 2016 and October 2020, 231 participants were consented and assessed for eligibility. Participants signed informed consent documents and completed eligibility and baseline assessments, including structured interviews and self-report measures. Randomization occurred after eligibility was determined. Randomization was accomplished in varying block sizes so that it would be difficult to guess the next random assignment. If participants ended treatment prematurely, they were asked to continue with assessments for intention-to-treat (ITT) analyses. In accordance with the Department of Defense Instruction pertaining to the protection of participants (DoDI 3216.02), participants were not paid for their involvement in the study or any of the assessments. Adverse events were monitored using methods similar to medication clinical trials.^17^ The full study protocol can be found in Supplement 1.

### Measures

All measures were administered by independent evaluators who were masked to treatment condition (eMethods 1 in Supplement 2). Assessments occurred at baseline and 10, 20, and 30 weeks after the first treatment session. A diagnostic assessment of PTSD according to the fifth edition of the *Diagnostic and Statistical Manual of Mental Disorders* (*DSM-5*)^18^ was completed using the Clinician-Administered PTSD Scale for *DSM-5* (CAPS-5) and repeated at subsequent assessments (ie, 10, 20, and 30 weeks post first treatment session). The CAPS-5 is a structured interview that assesses PTSD symptom severity and diagnostic status.^19^ Symptoms are rated on a scale from 0 (absent) to 4 (extreme/incapacitating). A total symptom severity score is calculated as the sum of the 20 symptom items, with a range of 0 to 80. Additional measures were also included in the assessment battery but are not described here, as they are not related to the primary study aims.^20^

### Treatment

WET^11^ consists of 5 weekly sessions. The first session is approximately 1 hour, and the remaining 4 sessions last approximately 45 minutes. In the first session, patients receive psychoeducational information about PTSD and the treatment rationale. Patients are then given specific instructions for writing about their trauma and write for 30 minutes. The therapist briefly checks in with the patient after the writing to determine whether there were any challenges in completing the writing. The same writing procedure is followed for the next 4 sessions.

CPT^10^ consists of 12 one-hour sessions that occur twice per week. CPT is a trauma-focused therapy in which patients are taught to recognize and challenge dysfunctional cognitions about their traumas and current thoughts about themselves, others, and the world. Patients learn to label events, thoughts, and emotions, while therapists help them examine the facts and context of the trauma through Socratic questioning. Using progressive worksheets, patients examine their thoughts and emotions and develop more balanced thinking about their traumas. Patients are also given assignments to complete between sessions. Training and fidelity of the therapists are described in eMethods 2 in Supplement 2.

### Statistical Analysis

#### Power Analysis

A power analysis was conducted with the focus being the test of noninferiority based changes in CAPS-5 PTSD severity scores. Following Sloan et al,^13^ we used an outcome difference of 10 points or more on the CAPS-5 total score as the noninferiority margin. Differences less than 10 points would be considered clinically insignificant, so noninferiority would be declared if the upper bound of the 95% 1-sided confidence limit of the difference between group means was less than 10. Using the CAPS based on criteria of the *DSM-IV*,^21^ Schnurr et al^22^ reported that the standard deviation of the CAPS to be 20, so this represents a standardized mean difference in terms of a Cohen *d *of 0.50, a conventional medium effect.^23^

Sample size was determined using the module for noninferiority tests in the NCSS/PASS power software. Specifications were a 10-point noninferiority margin on the CAPS-5, a standard deviation of 20,^22^ a true difference between treatment groups of 0, 1-sided noninferiority test at *P* = .05, desired power of 0.80, and equal allocation to the 2 treatment groups. With these specifications, PASS indicated that 50 participants per group was required. This number was increased by 25% to account for unavoidable loss to follow-up, which resulted in a target recruitment of 126 participants. This targeted sample size is consistent with other previously conducted PTSD noninferiority trials.^24,25^ Due to a higher than anticipated number of participants (17) who did not attend the first treatment session, we randomized a total of 169 participants.

#### Data Analytic Plan

We used mixed-effects regression models with repeated measures to examine noninferiority on CAPS-5 total scores. An advantage of likelihood-based regression models over conventional analysis of variance includes the ability to include all participants who completed baseline assessment regardless of the extent of study participation given the commonly accepted assumption that data are missing at random. Mixed-effects models consisted of fixed effects of time, intervention group, and their respective interaction. We calculated change score contrasts to examine PTSD severity reduction differences by intervention from baseline to 10-, 20- and 30-week assessments. As it was expected that WET would be noninferior to CPT, a 1-sided 95% CI was used for the noninferiority test of change from baseline to the 10-, 20- and 30-week assessments. Noninferiority was defined as the model-based difference between the 2 conditions being less than the upper bound of the 1-sided 95% CI specified margin of 10 points on the CAPS-5. Cohen *d* was calculated to further describe within- and between-group effect sizes using conventional interpretation guidelines.^23^

Analyses were completed using an ITT and a per-protocol (PP) sample of treatment completers. Existing research has shown that that PP analysis is an important supplement to complement the ITT analysis; however, PP alone is not recommended as an appropriate substitute.^26,27^ ITT analyses are widely assumed to yield a biased estimate favoring the null hypothesis of no differences, while analyses that only include the PP sample can be biased in either direction. Therefore, our criteria to accept the noninferiority hypothesis were based on the model-based difference being confirmed in both samples.

A generalized linear model evaluated overall dropout, and a Kaplan-Meier survival analysis examined timing of attrition for both 2 interventions. Participants who did not complete the 12 session protocol for CPT or 5 treatment session protocol of WET were considered treatment dropouts. For the survival analysis, time to event was defined as the number of sessions completed prior to dropout. The log-rank χ^2^ test pooled over strata was used to analyze group differences in the survival distribution. Given that CPT has more treatment sessions than WET, overall number of treatment dropouts was tested using the total session protocol and over the first 5 sessions, and time to dropout was examined using the first 5 treatment sessions for both interventions. All analyses were completed using SAS version 9.4 (SAS Institute) and SPSS statistical software version 26 (IBM Corp).

## Results

### Baseline Patient Demographics and PTSD Severity

Demographic characteristics by intervention condition and the total sample are presented in Table 1. Participants were predominantly male (136 [80.5%]) and served in the Army (167 [98.8%]), with a mean (SD) age of 34 (8) years.

### PTSD Noninferiority Analyses and Severity Outcomes

PTSD symptom severity descriptive statistics, outcomes, and results of noninferiority analyses for the ITT and PP samples are provided in Table 2 and Table 3. Within- and between-condition effect sizes are reported in Table 4. Both the ITT and PP samples demonstrated similar trends. Therefore, only the ITT outcomes are reported here unless otherwise specified. The primary end point per the research protocol indicated that WET was noninferior to CPT at all 3 postbaseline assessment periods on the upper limit of the 1-sided 95% CI for changes on the CAPS-5 being less than the 10-point noninferiority margin (Table 3). Mean (SE) differences in PTSD symptom reductions from baseline to 10, 20, and 30 weeks post first treatment session ranged from 0.33 (2.58) at week 30 to 3.96 (1.73) at week 10. Furthermore, the 1-sided 95% CI upper limit was less than the 10-point noninferiority margin across time points in both groups and ranged from 4.59 at week 30 to 6.81 at week 10. There were significant reductions in PTSD symptom severity across both treatment conditions and at all assessment periods, with medium to large within-group effect sizes. Between-group effect sizes in PTSD symptom severity change were all small (Table 4). Within-condition effect sizes ranged from a Cohen *d* of 0.48 for the WET group in the intention-to-treat analysis at week 10 to 0.95 for the CPT group in the per-protocol analysis at week 10, and between condition effect size ranged from 0.06 in the intention-to-treat analysis at week 30 to 0.22 in the per-protocol analysis at week 10.

**Table 2.** Clinician-Administered PTSD Scale for *DSM-5* Total Descriptive Statistics, by Intervention and Time^a^

Group	Participants, No./total No.	Score, mean (SE)			
		Baseline^a^	Follow-up		
			10-wk	20-wk	30-wk
**Intent-to-treat**					
WET	85/169	36.71 (1.12)	31.55 (1.13)	29.59 (1.45)	27.77 (1.61)
CPT	84/169	34.24 (1.13)	25.12 (1.40)	25.41 (1.49)	24.97 (1.68)
**Per protocol**					
WET	65/111	36.69 (1.32)	31.55 (1.40)	29.60 (1.53)	27.79 (1.69)
CPT	46/111	33.83 (1.57)	24.17 (1.70)	24.76 (1.72)	24.03 (1.85)

Abbreviations: CPT, cognitive processing therapy; *DSM-5*, *Diagnostic and Statistical Manual of Mental Disorders, Fifth Edition*; PTSD, posttraumatic stress disorder; WET, written exposure therapy.

^a^ Clinician-Administered PTSD Scale for *DSM-5* scores were not significantly different (*P* > .05) at baseline.

**Table 3.** Clinician-Administered PTSD Scale for *DSM-5 *Outcomes and Noninferiority Analyses

Group	Estimated score change score, mean (SE)		
	Baseline to 10-wk follow-up	Baseline to 20-wk follow-up	Baseline to 30-wk follow-up
**Intention to treat, WET vs CPT**			
WET	−5.16 (1.17)	−7.11 (1.53)	−8.93 (1.79)
CPT	−9.12 (1.28)	−8.83 (1.58)	−9.26 (1.86)
Difference, mean (SE)	3.96 (1.73)	1.71 (2.20)	0.33 (2.58)
UL of 1-sided 95% CI^a^	6.81	5.34	4.59
**Per protocol, WET vs CPT**			
WET	−5.15 (1.22)	−7.09 (1.64)	−8.91 (2.16)
CPT	−9.65 (1.50)	−9.06 (1.86)	−9.80 (1.64)
Difference, mean (SE)	4.51 (1.94)	1.98 (2.48)	0.89 (2.88)
UL of 1-sided 95% CI^a^	7.71	6.06	5.64

Abbreviations: CPT, cognitive processing therapy; *DSM-5*, *Diagnostic and Statistical Manual of Mental Disorders, Fifth Edition*; PTSD, posttraumatic stress disorder; UL, upper limit; WET, written exposure therapy.

^a^ Noninferiority was based on the UL of the 1-sided 95% CI being less than 10 points.

**Table 4.** Clinician-Administered PTSD Scale for *DSM-5* Treatment Outcome Effect Sizes, as a Function of Treatment Condition^a^

Group	Cohen *d*		
	Baseline to 10-wk follow-up	Baseline to 20-wk follow-up	Baseline to 30-wk follow-up
**Intention to treat**			
WET	0.48	0.54	0.54
CPT	0.78	0.61	0.54
Between-group difference	0.18	0.06	0.01
**Per protocol**			
WET	0.52	0.54	0.58
CPT	0.95	0.72	0.67
Between-group difference	0.22	0.08	0.03

Abbreviations: CPT, cognitive processing therapy; *DSM-5*, *Diagnostic and Statistical Manual of Mental Disorders, Fifth Edition*; PTSD, posttraumatic stress disorder; WET, written exposure therapy.

^a^ All within-condition effects were significant at *P* < .001.

We examined the percentage of participants who showed a reliable change in PTSD symptom severity, defined as a change of 12 points on the CAPS-5 (B.P. Marx, unpublished article, 2021). At 30 weeks, 12 of 32 participants (37.5%) in the CPT group and 17 of 36 (47.2%) in the WET group had a reliable change. This difference was not a statistically significant (χ^2^ = 0.66, *P* = .42).

### Adverse Events

We compared the frequency of adverse event (AE) reporting between the WET and CPT groups for visits 1 to 5, when the 2 groups were similarly scheduled, and for 3 three follow-up visits. During visits 1 to 5, 81 participants (54%) reported 121 AEs with approximately 20% reporting more than 1 AE. The most frequently reported AEs were psychiatric symptoms, such as anxiety, depression, and sleep disturbances. However, reports of pain, injury, illness, and various other medical problems accounted for most reports. Most AEs reported were not determined to be related to treatment; 36 AEs were considered to be related to the study procedures, including the therapies administered.

Although overall AE reporting did not differ between the two groups, the WET group had more AEs judged to be study-related, and anxiety was the most commonly reported AE. During the follow-up period, fewer participants reported fewer AEs, with no group differences, and only 1 was judged to be study-related. During CPT visits 6 to 12, 27 participants (47%) reported a total of 46 AEs, with 8 of those being PTSD-type symptoms considered to be study-related.

### Survival Analysis

The total sample attrition was 33.7% (57 participants), with 17 (10.1%) never starting treatment. There was a significant between-group difference in overall dropout (χ^2^_1_ = 8.83; *P* = .003). Participants randomized to CPT were more likely to drop out of treatment (dropout rate, 45.2% [37 participants]; OR = 2.69 [95% CI, 1.39-5.20]) relative to participants randomized to WET (dropout rate = 23.5% [20 participants]). When treatment completion was defined as attending at least 5 sessions, attrition decreased to 23.7% [20 participants] and 23.8% [20 participants] in WET and CPT, respectively. Using this method, there were no significant group differences in attrition (χ^2^_1_ = 0.002; *P* = .97) and time to attrition (χ^2^_1_ = 0.01; *P* = .93). Participants who dropped out of treatment were more likely to not return for any postbaseline assessment (χ^2^_1_ = 32.151; *P* < .001).

## Discussion

Despite having fewer than half as many treatment sessions, WET was noninferior to CPT in the treatment of PTSD among active- duty service members. Between-condition effect sizes were less than 0.20 at all follow-up assessments, and the noninferiority findings were observed with both the ITT and PP analyses. PTSD symptom severity was significantly reduced in both treatment conditions, with medium to large within-condition effect sizes. Not surprisingly, PTSD symptoms continued to improve over time. This is notable given that the first postbaseline assessment occurred just 10 weeks after the first treatment session. At that time, all but 7 participants assigned to CPT had completed treatment.

Consistent with results from prior studies,^13,28,29^ these findings support the notion that PTSD can be treated effectively with fewer treatment sessions than previously thought. The efficiency of WET has particular value for the military setting, where the time to receive PTSD treatment may be limited due to several factors. The efficiency of WET pertains not only to the total number of treatment sessions but also to the fact that there are no between-session assignments and less time is needed for provider training.^11^

The reduction in PTSD symptoms is similar to what has been observed in other treatment studies with service members and is consistent with the general finding that service members with PTSD may be more challenging to treat than civilians.^30^ The standard error found in this study was large for both interventions, reflecting considerable variability in treatment response across service members. This pattern may underscore the importance of focusing on who may and may not benefit from PTSD treatment. Unfortunately, we are underpowered to investigate possible moderators of outcomes. The heterogeneity in PTSD treatment responses has been noted by others as well^31^ and should be a focus of future PTSD research.

We initially found that WET had almost half as many treatment dropouts (24%) than CPT (46%). However, we found no differences in dropout when limiting our analyses to the first 5 sessions. Treatment dropout occurred consistently throughout the course of CPT; this finding contrasts with observations from other studies in which most CPT treatment dropout occurred early in treatment.^13^ Studies that have observed dropout to be most likely early in CPT included the written accounts component of the protocol,^32^ raising the possibility that the assignment to complete a trauma narrative between sessions may have resulted in increased early dropout. One potential solution to reduce CPT dropouts is to conduct daily, massed CPT sessions over a much shorter period of time, such as a week, to encourage completion of treatment quickly.^33^

Study strengths include a large, racially diverse sample. Although women service members represented a small proportion of the sample (20%), their representation is consistent with the percentage of women in military service. The study also structured assessments to occur at the same time for participants in both treatment conditions so that time to complete treatment would not serve as a potential treatment outcome confound. Nevertheless, all but 7 participants completed treatment by the 10-week assessment. The 20- and 30-week assessments, therefore, served as a proxy follow-up assessment period. Examination of noninferiority at both the approximate posttreatment assessment and subsequent assessments provides a greater examination of noninferiority of both treatment outcome and maintenance of treatment gains.

Our findings from this study of a military sample are consistent with those observed for a primarily civilian sample.^13^ These consistent findings across samples suggest our results are generalizable.

### Limitations

This study has limitations. Not surprisingly, participants who dropped out prematurely were less likely to return for subsequent assessments. Another potential limitation is the lack of long-term follow-up, which precludes us from knowing whether treatment gains were maintained beyond 6 months post treatment.

## Conclusions

In this study, WET and CPT both significantly reduced PTSD symptom severity among service members to similar degrees, even though CPT is more time- and resource-intensive. Overall, fewer participants dropped out of WET than CPT. The option of a brief PTSD treatment is likely to be of high value in the military setting, where military service operations may limit treatment engagement. One clear pattern of findings in this study is the high variability of treatment outcome among service members. Better understanding of the factors associated with who does and who does not benefit from PTSD treatment is an important direction for the field.

## Notes

Supplement 1.
Trial Protocol

Supplement 2. **eMethods 1. **CAPS-5 Protocol **eMethods 2. **Therapists

Supplement 3.
Data Sharing Statement
