# Supplementary material for: Effect of Written Exposure Therapy vs Cognitive Processing Therapy on Increasing Treatment Efficiency Among Military Service Members With Posttraumatic Stress Disorder: A Randomized Noninferiority Trial
Source: JAMA Netw Open. 2022 Jan 11;5(1):e2140911. doi: 10.1001/jamanetworkopen.2021.40911 (PMC8753496; doi:10.1001/jamanetworkopen.2021.40911)
Supplement: Supplement 1. — Trial Protocol [file jamanetwopen-e2140911-s001.pdf]

HUMAN SUBJECTS RESEARCH PROTOCOL

1. **PROTOCOL TITLE:** Brief treatment for Posttraumatic Stress Disorder: Enhancing treatment engagement and retention

2. **ABSTRACT:** The goal of this randomized clinical trial is to investigate if a brief, written intervention for posttraumatic stress disorder (PTSD), Written Exposure Therapy (WET), is equally efficacious as an evidenced-based behavioral therapy, Cognitive Processing Therapy-Cognition only (CPT-C), in the treatment of PTSD in active duty military men and women with a diagnosis of PTSD The WET condition consists of 5 weekly sessions, with the first session requiring one hour and the remaining four sessions requiring approximately 40 minutes. CPT-C consists of 12, one hour sessions that will take place twice per week. This study is designed to determine if WET is as equally efficacious (i.e., noninferior) to CPT-C, in reducing PTSD symptoms The primary study outcome will be change in symptom severity as assessed by the Clinician Administered PTSD Scale for DSM-5 (CAPS-5). Independent assessors will evaluate participants using the CAPS-5 at baseline,10, 20-, and 30-week intervals after the start of treatment.

3. OBJECTIVES/SPECIFIC AIMS/RESEARCH QUESTIONS

Primary Aim: To conduct a RCT to examine if WET is noninferior to CPT-C for the treatment of PTSD in active duty military personnel.

- **Hypothesis 1**: Participants randomly assigned to WET will show noninferior change in PTSD symptom severity at the 10-week assessment relative to participants randomly assigned to CPT-C.
- **Hypothesis 2**: Participants randomly assigned to WET will show noninferior outcomes in PTSD symptom severity at the 20- and 30-week assessment relative to participants randomly assigned to CPT-C.

Secondary Aim: Examine whether there are condition differences in treatment dropout rates between WET and CPT-C.

- **Hypothesis 3**: WET will have a significantly lower dropout rate relative to CPT-C.

4. **MILITARY RELEVANCE:** Many military personnel are reluctant to seek treatment from the military treatment facilities and when they do seek out treatment, treatment dropout rates can be high (1). Hoge and colleagues (1) described the current state of treatment utilization and dropout among military personnel as a “call to action to improve treatment engagement and retention” (p. 997). The proposed project addresses this important and much needed area.

5. BACKGROUND AND SIGNIFICANCE.

Given the high prevalence of PTSD among military personnel, the deleterious consequences associated with PTSD when left untreated, and the concerns about the patient engagement and utilization as well as clinician adherence and implementation of Prolonged Exposure (PE) and Cognitive Processing Therapy (CPT), there is an urgent need to identify alternative PTSD treatment approaches that are more efficient, more accepted and tolerable to patients, and perhaps more amenable to dissemination within a military context. Ideally, such an alternative treatment approach would also involve less training to implement and would increase provider adherence. One potential solution is narrative therapy, which involves repeatedly confronting a trauma memory through writing. Several narrative therapy protocols have been tested and have been associated with low treatment dropout and high client treatment satisfaction rates (2). Moreover, narrative therapy can be successfully implemented by peer counselors (3), and continues to be effective even when a flexible protocol version is used (3). Although Hoge (4) suggested that narrative therapy may be a viable treatment alternative for military-related PTSD and the most recent Agency for Healthcare Research and Quality (AHRQ) PTSD effectiveness report (5) included narrative therapy as an effective PTSD treatment, this treatment approach has not yet been tested in active duty military service men and women with PTSD.

There are several different available narrative writing protocols that have been used to treat PTSD (2). One narrative therapy protocol is called written disclosure (WD). In a series of studies examining participants with a trauma history and at least moderate PTSD symptom severity, Sloan and colleagues found that, relative to a control writing condition in which participants were instructed to write about emotionally neutral experiences, writing about traumatic events significantly reduced PTSD symptom severity (6,7,8). However, when including participants who had a diagnosis of PTSD, WD was not associated with significantly reduced PTSD symptom severity relative to a control writing condition (9). Importantly, the results also showed that participants assigned to the WD condition did not experience the significant reduction in arousal

and negative affect from the first to the last session. These findings suggested that the therapeutic dose (three, 20 minute writing sessions) may not have been sufficient to produce significant benefits in participants with a PTSD diagnosis. Other possible explanations for the null findings included the fact that study participants were not provided with any treatment rationale or psychoeducation about PTSD. Past research has suggested that these components may be necessary, but not sufficient, for successful treatment outcomes (e.g., 10).

With these findings in hand, Sloan and colleagues altered the treatment protocol in several important ways. First, treatment rationale and psychoeducation components were added to the first treatment session. Next, based on prior work indicating the importance of directing individuals to write repeatedly about the details of their index trauma, with particular attention to felt emotions, the meaning of the traumatic event, and "hot spots" (7,8), significant modifications to the writing instructions were made. To reflect these changes and distinguish the original Pennebaker and Beall (11) WD protocol from the modified protocol, the current protocol is referred to as written exposure therapy (WET). Importantly, in the WET protocol, there are no between-session assignments included and time spent with a therapist is minimal as the therapist merely reads the writing instructions to the individual and then leaves the person alone to complete the 30 minutes of writing. The WET protocol was designed to be consistent with the goal of creating a tolerable and efficient exposure-based treatment alternative for PTSD. The minimal therapist contact, in combination with the minimal time needed to train therapists to implement the treatment, results in an approach that addresses many of the difficulties associated with using PE and CPT in Department of Defense (DoD) and Department of Veteran Affairs (VA) mental health clinics.

An efficacy study of WET as an intervention for motor vehicle accident (MVA)-related PTSD was conducted. In this RCT, participants were randomized to either WET (n = 24) or wait-list (WL; n = 22). Median time since MVA was 20 months and all of the participants were diagnosed with chronic PTSD using a structured diagnostic interview. Participants that were randomized to WET displayed a large and significant reduction in PTSD symptoms at post-treatment and 3 month follow-up, relative to participants in the WL. WET participants also maintained their treatment gains at the 6 month follow-up assessment. In terms of PTSD diagnosis, at the 3-month follow-up assessment only 4% of the WET participants met diagnostic criteria for PTSD, relative to 67% of the WL participants; 8% of WET participants met PTSD criteria at the 6 month assessment. Although no *in vivo* exposures were included in the WET protocol, the WET participants reported significant reductions in driving and riding avoidance behaviors at the follow-up assessments (15 point reduction for WET compared with 3 point reduction for WL; 12). Not only was WET efficacious in terms of reducing PTSD symptoms, the treatment was also well received and tolerated. Only 2 participants (8%) dropped out of the WET condition. The 8% dropout rate is consistent with the low dropout rates observed in earlier work (6,7,8). This treatment dropout rate compares favorably with the dropout rates reported for PE and CPT, which again are typically around 25-35% (13). The reason for the low dropout rate in WET is unclear but the limited number of sessions in combination with the current study will further extend our investigation of the efficacy of WET by comparing it directly to a first line PTSD treatment, Cognitive Processing Therapy-Cognition only (CPT-C) and testing it in an active duty military sample.

6. **<u>RESEARCH DESIGN</u>.** We will randomize up to 175 active duty military personal who meet diagnostic criteria for PTSD. Participants will be blocked randomized to either WET or CPT-C. Participants will be assessed at baseline (pre-treatment) and 10- , 20-, and 30- weeks following the baseline assessment. Primary outcome measure will be PTSD symptom severity at the assessment time points. PTSD symptom severity will be assessed using the Clinician Administered PTSD Scale for DSM-5 (CAPS-5). Treatment will consist of five weekly sessions for WET condition and twelve twice-weekly sessions for CPT-C.

7. <u>RESEARCH PLAN</u>

7.1 Selection of Subjects

7.1.1. Subject Population. The target population is 275 active duty service men and women who are at least 18 years old, and meet diagnostic criteria for PTSD.

7.1.2. Source of Research Material. All measures are being administered for research purposes.

**Measures**	Baseline	Weekly During Treatment	End of Treatment	10 weeks	20 weeks	30 weeks
**Demographic Information**						

1. Demographics & Military Service Characteristics	x					
**PTSD Measures**						
2. Life Events Checklist-5 (LEC-5)	x			x	x	x
3. Deployment Risk and Resilience Inventory (DRRI-2) Combat Experience and Postbattle Experience Sub-Scales if applicable	x					
4. *Clinician Administered PTSD Scale (CAPS-5)	x			x	x	x
5. PTSD Checklist for DSM-5 (PCL-5)	x	x		x	x	x
**Sleep Measures**						
6. Insomnia Severity Index (ISI)	x			x	x	x
7. Snoring, Tired, Observed, Blood Pressure (STOP) Sleep Apnea Screen	x					
8. PROMIS Sleep Disturbance and Sleep-Related Impairment	x			x	x	x
**Health Measures**						
9. History of Head Injuries	x			x	x	x
10. Veterans Rand 12-Item Health Survey (VR-12)	x			x	x	x
11. Fagerstrom Test for Nicotine Dependence (FTND)	x			x	x	X
12. Fagerstrom Test for Nicotine Dependence – Smokeless Tobacco Version (FTND-ST)	x			x	x	x
13. Health Questionnaire	x			x	x	x
**Other Psychosocial Measures**						
14. *Mini International Neuropsychiatric Interview (MINI 7.0) - Psychotic Module	x					
15. Patient Health Questionnaire (PHQ-9)	x	x		x	x	x
16. Depressive Symptom Index – Suicidality Subscale (DSI-SS)	x			x	x	x
17. *Self-Injurious Thoughts and Behaviors Interview (SITBI)	x			x	x	x
18. Generalized Anxiety Disorder Screener (GAD-7)	x			x	x	x
19. Alcohol Use Disorders Identification Test (AUDIT)	x					
20. Quick Drinking Screen (QDS) self-report version	x			x	x	x
21. Brief Inventory of Psychosocial Functioning (B-IPF)	x			x	x	x
Therapy Process Measures						
22. Credibility Expectancy Scale (CEQ) Pre and Post	x		x			
23. Client Satisfaction Questionnaire (CSQ)			x			
24. Working Alliance Inventory-Short Revised (WAI-SR)			x			

* clinician-administered interviews

7.1.3. Inclusion and Exclusion Criteria.

Inclusion Criteria:

- Adult (18 years or older) male and female active duty military personnel seeking treatment for PTSD
- Diagnosis of PTSD determined by Clinician Administered PTSD Scale for DSM-5 (CAPS-5)
- Ability to speak, read and write English
- Not currently engaged in psychosocial treatment for PTSD
- Individuals taking psychotropic medications agree to work with their prescriber to remain on stable doses of any prescribed psychotropic medications for the duration of the intervention and through the first follow-up assessment as much as possible and as medically indicated.

Exclusion Criteria

- Current suicide or homicide risk meriting crisis intervention as determined by Depressive Symptom Index – Suicidality Subscale (DSI-SS) and Patient Health Questionnaire-9 (PHQ-9)

- Active psychosis as determined by the psychosis module of the Mini International Neuropsychiatric Interview (MINI) interview
- Moderate to severe brain damage (as determined by the inability to comprehend the baseline screening questionnaires)

7.1.4. Description of the Recruitment and Prescreening Process. Potential participants will be recruited in various ways including self-referring in response to STRONG STAR billboards and advertisements posted on the STRONG STAR website and social media. IRB-approved recruitment flyers (See Appendix C) will be posted and disseminated across the San Antonio, TX and Killeen, TX communities at places frequented by active duty Service Members. For example, recruitment materials will be distributed to Fort Sam Houston, Lackland Air Force Base, and Fort Hood primary care locations as well as on-post/base behavioral health clinics, Soldier/Airman & Family Centers, fitness centers, chapels, barracks, military exchanges, and other places Service Members frequent. Furthermore, potential participants can be recruited through referrals from health care providers at the military clinics. At Ft Hood, providers can also refer interested individuals directly to STRONG STAR using the hospital's electronic referral system. Research staff will discuss the study treatment and eligibility requirements with potential participants (See Appendix D; Pre-Screen Phone Script). If staff believe a person may qualify for the study, an appointment will be made for consent and screening.

7.1.5. Consent Process. An authorized and trained member of the research team will engage the potential participant in an interactive explanation of the study guided by the informed consent document (ICD). After the participant has read the ICD, he or she will be given the opportunity to consider participation and discuss the research with family and friends. Once the potential participant has reached a decision, the advising staff member will review the purpose of study, duration of study, study procedures, the experimental components of the study, the potential risks and discomforts, the potential benefits, any alternatives to participation, protection of participant's confidentiality, and the contact information for both the researchers and the regulatory bodies overseeing the conduct of the study with the participant to ensure the participant has an understanding of the study. If the individual is agreeable to participation the advising staff member will then have the individual sign the consent form in the presence of a witness. A copy of the signed ICD will be given to the participant for their reference. Over the conduct of the study, the research team will be available to answer any questions about the research. Ongoing discussions will occur to ensure the participant's questions and concerns are addressed during the conduct of the study. Potential participants will have the study explained to them in a safe and private location before any assessments are conducted within the STRONG STAR offices in San Antonio or STRONG STAR offices in Killeen, TX. The informed consent process will require approximately 20 minutes.

7.1.6. Subject Screening Procedures. Once the consent is signed, participants will then be asked to fill out the packet of assessments (see measures section below) with the Independent Evaluator (IE). The initial consent and screening will require 3-4 hours.

7.1.7. Compensation for participation. N/A.

7.1.8. Treatment Procedures. Participant will be randomized to one of two treatment conditions.

- Written Exposure Therapy (WET): WET consists of five sessions (meeting once weekly), with the first session requiring approximately one hour and the remaining four sessions consisting of approximately 40 minutes. In the first session, participants will be provided with psychoeducation information about PTSD and treatment rationale. Instructions for writing about the traumatic event are then provided. Participants are then left alone to write about their trauma event for 30 minutes. The therapist returns after 30 minutes and checks in briefly with the participant to see how the writing went. The next four sessions the participant is provided with the specific writing instructions for that day and then left alone to complete the 30 minute writing session. The therapist then returns after 30 minutes to prompt the participant to stop writing and check in about how the writing went during that session. See Appendix A for WET manual.

- Cognitive Processing Therapy-cognition only (CPT-C): CPT-C consists of 12, one hour sessions that occur twice per week CPT-C consists of challenging trauma-related cognitions (e.g., I am to blame for what happened to me") with the goal of changing maladaptive cognitions to more adaptive cognitions. In addition to attending treatment sessions, participants will be given assignments to be completed between treatment sessions. See Appendix B for CPT-C manual.

7.2 **Drugs, Dietary Supplements, Biologics, or Devices*.*** N/A

7.3. Study Procedures/Research Interventions. The following study procedures will be followed:

- A member of the Research Team will explain the purpose of the study to potential participants. Potential participants will be asked to read and sign the consent.
- If the participant has been referred from another STRONG STAR study and already undergone baseline testing, the participant will be asked as part of the consent process to use these assessments rather than repeating the assessment battery.
- If the participant is newly referred to this study or declines use of previously completed assessments, he or she will meet with an evaluator and complete the assessment questionnaires.
- The results of these screening procedures will be reviewed to determine whether it is appropriate for the participant to continue in the study. If it would not be appropriate for the person to continue in the study, a member of the Research Team will discuss the reasons why and, if needed, coordinate appropriate follow-up outside of this study.
- Once all the baseline testing is completed, the participant will be randomized to either Written Exposure Treatment (WET) or Cognitive Processing Therapy – Cognition only (CPT-C) and scheduled to start treatment in accordance with the condition they were randomized into. Randomization will be stratified to consider gender and PTSD symptom severity baseline score. Randomization will be done using a table of random numbers.
  - Written Exposure Treatment (WET). Participants will complete 5, weekly sessions during which they will be instructed to write about their trauma event for 30 minutes each session. The first session lasts approximately 60 minutes, whereas the remaining four sessions require approximately 40 minutes to complete.
  - Cognitive Processing Therapy – Cognition only (CPT-C). Participants will complete 12, 60 minute sessions that will be scheduled twice per week. Treatment focuses on challenging traumatic-related cognitions.
- Every effort will be made to deliver the therapy in the therapist's office face-to-face. However, in the event that a therapist is unavailable on-site, a therapist trained in the interventions and located in Texas will provide therapy by video teleconferencing. Video teleconferencing will be accomplished using the UTHSCSA stand-alone network that allows for platform-based encryption. The participant will be located in the STRONG STAR offices at Ft Hood using a UTHSCSA computer and the therapist will be located in their office also using a UTHSCSA computer. Platform-based encryption technology has been recognized as superior to enterprise networks for data security (Younggren, 2011) and the stand-alone network allows for full control of scheduling and bandwidth for the research. For this research we will use Cisco Systems C20 Quickset Telepresence System to create a stand-alone VTC network allowing full control over scheduling and bandwidth use. The C20 system (also referred to as MOVI or Jabber) meets all of the technical specifications outlined in the ATA Guidelines, and is easy enough to use that even technical novices should be able to participate successfully in VTC mental health. Telepresence through the C20 Quickset is encrypted and cloud-based allowing for easy access to any individual with a personal computer and high-speed internet access. Because it is cloud-based (i.e., the encrypted and HIPAA secure encounter occurs on the internet instead of on a closed system).
- Follow-up assessments will be completed 10-, 20-, and 30-weeks following the baseline assessment. All study participants will be on-study approximately 8 months.

7.3.1 Collection of Human Biological Specimens. N/A

7.3.1.1 Laboratory evaluations and special precautions. N/A

7.3.1.2 Specimen storage*.* N/A

7.3.2 Data Collection.

7.3.2.1 Instrumentation. See the table at Section 7.1.2 above for a summary of the assessments and timing of administration. All measures are being administered for research purposes. A description of each of the assessments can be found at the end of this protocol. Assessments will be administered in person whenever possible. However, in order to accommodate participant schedulesand/or instances in which a participant may have left the local area at the time of a follow up assessment, we may collect full or partial assessments in person or via phone or video teleconference or electronic data capture using a secure link to the encrypted STRONG STAR database. Reasonable efforts will be made to collect all data as described in this protocol, but we expect some participants may not be able to complete part or all of any given follow up assessment.

7.3.2.2 Data Storage and Access. Data will be coded using an assigned number. Hard copies of data collected during treatment will be placed into a lock box which will be transported by car to University of Texas Health Sciences Center San Antonio (UTHSCSA) STRONG STAR offices by a STRONG STAR staff member who will place it into the locked cabinets at the STRONG STAR offices. Data will be entered into the STRONG STAR database on a secure server by member of the research team. Electronic data will be stored, managed, and analyzed by the Data Management and Biostatistics Core staff of the STRONG STAR consortium. The overall PI and named collaborators will have access to identifiable data through the STRONG STAR website and UTHSCSA server. Every member of the research team will be trained and monitored about how to handle and protect both medical and research records. Furthermore, the research team strictly controls access to study data.

All UTHSCSA STRONG STAR network connectivity is segmented with Access Control Lists and is not accessible to any other UTHSCSA network segments. STRONG STAR data server is physically located at the Advanced Data Center (ADC) has 24x7 onsite security, card key, biometric access controls and video surveillance. University of Texas Health Science Center at San Antonio (UTHSCSA) ADC facility also maintains Gen 2 firewall devices to protect and prohibit any unauthorized access to UTHSCSA data. All UTHSCSA network devices are monitored by state of the art monitoring applications that include configuration audit, management, and availability 24x7.

The UTHSCSA STRONG STAR data server is currently a VMware Instance running Windows Server 2016 Enterprise Standard with daily backup services and vSphere Business Continuity Advanced Failover.

Only select Data Core personnel have direct access to the data on a “need to access basis”. Data Core also follows the Principals Of Least Privilege (POLP). For example (but not limited to) detecting and repairing data corruption and producing reports not currently within the STRONG STAR system. All user activity is tracked and recorded within the system so if any records are added, altered or viewed the action is recorded and can be recalled for auditing purposes.

7.3.3. Human Biological Specimen (Biomarker) Processing. N/A

7.4 Statistical Consideration

7.4.1 Sample Size Estimation. Following the practice of Schnurr et al. (14) and Monson (15, described in 16), an outcome difference of 10 points or more on the CAPS total severity score was chosen as the “non-inferiority margin.” Differences smaller than 10 points would be considered clinically insignificant, so non-inferiority will be declared if the upper bound of the 95% one-sided confidence limit of the difference between group means is less than 10. Schnurr et al. reported the standard deviation of the CAPS to be “roughly 20,” so this represents a standardized mean difference in Cohen’s terms (17) of d=.50, a conventional medium effect.

Sample size was determined using the appropriate module for non-inferiority tests in the NCSS/PASS power software (18). Specifications were a 10 point non-inferiority margin, a standard deviation of 20 (14), a true difference between treatment groups of zero, one-sided non-inferiority test at p=.05, desired power=.80 and equal allocation to the two treatment groups. With these specifications, PASS reports that N=50 per group is required (note: the same result is given by several free online calculators, e.g., https://www.sealedenvelope.com/power/continuous-noninferior/; http://powerandsamplesize.com/Calculators/Compare-2-Means/2-Sample-Non-Inferiority-or-Superiority). As noted in the application, this number was increased twice, first by 25% to account for unavoidable loss to follow-up, and then by an additional 20% to deal with the as yet unknown psychometric properties of the CAPS-5. This is the basis for proposed recruitment of N=175.

Estimate Required Sample Size	**Up to 175**
Estimate Participant Screen Out / Drop Out / Withdrawal	**30%**
Total Enrollment Requirement	**275**

7.4.2 Primary (i.e., primary outcome variables) and secondary endpoints.
The primary treatment outcome variable in this study is PTSD symptom severity, which is assessed using the CAPS-5. The

CAPS-5 will be administered at baseline, 10-, 20-, and 30- weeks following the baseline assessment. The secondary outcome variable is treatment dropout rate for each treatment condition.

7.4.3 Data analysis.

Hypothesis 1: Participants randomly assigned to WET will show noninferior outcomes in PTSD symptom severity at the 10-week assessment relative to participants randomly assigned to CPT-C.

Hypothesis 2: Participants randomly assigned to WET will show noninferior outcomes in PTSD symptom severity at the 30-week follow-up assessment relative to participants randomly assigned to CPT-C.

We will conduct analyses on both the modified intent to treat (ITT) sample (requiring only attendance at one treatment session) and on the per-protocol (PP) sample of treatment completers. Several authors (e.g., 16, 19) note that PP analysis is an important supplement to the ITT analysis but not a substitute. Although ITT analyses are widely assumed to have a bias in favor of the null hypothesis of no difference, systematic reviews do not always confirm that. Analyses of the PP sample may also be biased, and in either direction. At this point, we believe the consensus in the field is to perform analyses on both ITT and PP samples, and accept the non-inferiority hypothesis only if it is confirmed in both samples.

Random effects linear (hierarchical linear) models will be implemented for the primary noninferiority ITT randomized comparison of total CAPS-5 score as well as any secondary analyses. This ITT comparison will disregard all CPT-C non-adherence occurring after randomization under the ITT principal (20). The random effects linear models will consist of a random intercept and slope to account for within-patient correlations for the longitudinal observations across follow-up visits (baseline to 10-, 20-, and 30-weeks assessments). Fixed effects specified separately for each post-baseline visit, the intervention, and their respective interactions will be used to obtain the ITT estimate and one-sided 95% confidence interval for the noninferiority test of change from baseline at the 10 week assessment. The test of noninferiority will be based on showing that this upper bound of the one-sided confidence interval is less than the pre-specified margin of 10 CAPS points that is considered to show that WET is noninferior to CPT-C. The 10 week visit will be used to test noninferiority (**Hypothesis 1**), as this would be the first time point of post-treatment for both treatment conditions. Noninferiority will be claimed if the model-based difference between the two conditions is less than this upper bound. The 30 week post-baseline assessment will be used to examine **Hypothesis 2**.

Hypothesis 3: WET will have a significantly lower dropout rate relative to CPT-C.

We will conduct survival and logistic regression models to examine treatment dropout rates between the two conditions. The regression models can incorporate predictors (covariate main effects) of dropout, model the timing of attrition, and explore interactions to supplement the primary test of group differences. We will only classify a participant as a dropout if they did not complete the treatment protocol and dropped out of the treatment for reasons other than feeling better and not needing additional treatment.

7.7 Confidentiality. All in-person therapy sessions and interview assessments will be delivered in private offices in the STRONG STAR offices at the university Northwest Center located at 7550 IH10 West, Suite 1325 or STRONG STAR offices located at 4201 W Stan Schlueter Loop in Killeen, TX. Digital audio recordings of assessments will be labeled with the participant's study id number and saved on a secure password protected server. Those recordings to be reviewed for fidelity to ensure that the treatment is being delivered in accordance with the treatment manual will be viewed on a secure password protected server. There is no option for the reviewers to download or otherwise save the recordings to their computers. Every member of the research team will be trained and monitored about how to handle and protect both medical and research records. Only authorized study staff, and members of the STRONG STAR Data Management and Biostatistics Core staff will have access to either the raw data or electronic study data.

7.7.1 Certificate of Confidentiality. We are not seeking a Certificate of Confidentiality

7.7.2. Data Protection. Data will be coded using an assigned number. Paper copies of data collected during treatment will be placed into a lock box which will be transported by car to STRONG STAR offices by a STRONG STAR staff member. Data collected during treatment will be placed into locked cabinets at the UTHSCSA STRONG STAR offices at the Northwest Center in San Antonio or the STRONG STAR offices on Fort Hood by a STRONG STAR staff member and then entered into a secure STRONG STAR database. Audio files will be uploaded to a secure STRONG STAR server over an encrypted network connection.. Every member of the research team will be trained and monitored about how to

handle and protect both medical and research records. Furthermore, the research team strictly controls access to study data using policies and procedures developed specifically for the STRONG STAR Research Consortium.
.

A Data Safety and Monitoring Plan (DSMP) has been developed in accordance with the National Institutes of Health Office of Human Research Protection to assure the appropriate clinical safety monitoring of study subjects participating in this study.

8.0 RISKS/BENEFITS ASSESSMENT

8.1 Risks.

Likely but not Serious (expected to occur in more than 1 in 5 participants):

- Possibility of becoming emotionally upset or experiencing an initial increase of PTSD symptoms due to the discussion or journaling of traumatic events.

Rare and Serious (expected to occur in less than 5 out of 100 participants):

- With the handling of medical and research records there is always the possibility of a breach of confidentiality. However, every effort is made to protect the privacy of participants. Every member of the research team is carefully trained and monitored about how to store, handle, and protect participant records.

Risks of PTSD Diagnosis regardless of Treatment:

- Possibility of increased suicidal risk. One of the risks of PTSD both in and out of treatment is attempted suicide, which can result in death.

Safeguards for Protecting Participants.

During the early sessions of treatment, participants will be provided immediate coping tools and techniques used to manage distressing emotions by the study therapist. Distress experienced by participants is expected to be temporary. Any indication that the participant is considering suicide will be handled following care facility SOPs and using processes developed by military and civilian consultants to the STRONG STAR Consortium. Trained clinicians and evaluators will assess history of suicide and current suicidal ideation using the standardized measures such as the Depressive Symptoms Index – Suicidality Subscale or the Patient Health Questionnaire-9 (PHQ-9) and the Self-Injurious Thoughts and Behaviors Interview short form. For participants identified as having low to moderate risk for suicide based on the assessment results, the patient will be maintained on protocol and additional risk management procedures will be implemented within the context of the study treatment. For participants identified as being at high risk for suicide based on the assessment results, disenrollment will be considered if it is unlikely that standard treatment plus additional risk management procedures will maintain safety. High risk participants who are disenrolled from the study will be referred for more intensive treatment.
Risk of loss of identifiable information will be addressed by using unique identifiers for participant data. See above Section 7.7.2., Data Protection, for further details on protecting data and maintaining confidentiality.

Research Monitor: In addition, the study Research Monitor will oversee the safety of the research and report observations/findings to the IRB or a designated institutional official. The Research Monitor will review all unanticipated problems involving risks to subjects or others associated with the protocol and provide an independent report of the event to the IRB. The Research Monitor may discuss the research protocol with the investigators; shall have authority to stop a research protocol in progress, remove individual human subjects from a research protocol, and take whatever steps are necessary to protect the safety and well-being of human subjects until the IRB can assess the monitor's report; and shall have the responsibility to promptly report their observations and findings to the IRB or other designated official and the HRPO.

8.2 Potential Benefits. Potential benefits of participation in this study may include a reduction in PTSD symptoms over the course of therapy. Our primary goal is to treat participants to the point of symptoms reduction below the level of diagnostic criteria for PTSD. In addition, the knowledge gained from this study will serve to inform the most effective early interventions for the prevention and treatment of PTSD.

8.3 Alternatives: Mental health treatment is available at the San Antonio Military Medical Center at both the Brooke Army Medical Center (BAMC) and at the Wilford Hall Ambulatory Surgical Clinic (WHASC) in San Antonio, TX or the Carl. R. Darnall Army Medical Center (CRDAMC) in Killeen, TX including various forms of psychotherapy and drug treatments. Service Members can request treatment for PTSD through Army One-Source and may be eligible for care at one of the Veterans Healthcare System facilities or clinics. Not participating in the study is also an alternative.

9.0 ADVERSE EVENTS, UNANTICIPATED PROBLEMS, AND DEVIATIONS

9.1 Adverse Events will be assessed and monitored according to the established STRONG STAR SOP and the treatment facility's policies and procedures.

9.2 Reporting Adverse Events, Unanticipated Problems Involving Risks to Subjects or Others (UPIRSOs), and Deviations to the Office of the IRB.

All adverse events, unanticipated problems involving risk to subjects or others, and deviations will be reported to the Institutional Review Board (IRB) in accordance with current IRB policy. UPIRSOs and recurrent non-compliance with study procedures will be reported promptly to the IRB. Further, the study Research Monitor will review all unanticipated problems involving risks to subjects or others associated with the protocol and provide an independent report of the event to the IRB. All adverse events that do not meet the UPIRSO criteria and deviations that are not non-compliance will be summarized at Continuing Review per the IRB of record's policy.

10.0 WITHDRAWAL FROM STUDY PARTICIPATION. Participants may withdraw themselves from this study at any time and for any reason. Withdrawal from this study does not affect the participant's eligibility for care or any other benefits to which entitled. Participants who request to discontinue treatment, but who do not choose to withdraw from the study completely, will be asked to return for the post-treatment assessments. If a participant stops attending treatment sessions without notifying research staff, the therapist or project coordinator will make diligent attempts to contact the person to evaluate their status, attempt to re-engage them in the treatment, and encourage them to complete follow up assessments. Research staff will also refer to appropriate outside resources if necessary. Investigator may choose to withdraw a participant after consultation with the treating therapist and other consultants as appropriate in instances not limited to:

- Patient is noncompliant with treatment requirements
- Patient is in need of more intensive treatment
- Patient's symptoms worsen significantly
- Patient experiences a serious adverse event that is clearly related to the treatment
- Patient becomes actively suicidal
- Unexpected unavailability of a treating therapist

11.0 USAMRMC Volunteer Registry Database. We do not anticipate that this will be a greater than minimal risk study necessitating the use of the Volunteer Registry Database will be necessary.

12.0 REFERENCES.

1. Hoge, C. W., Grossman, S. H., Aucherlonie, J. L., Riviere, L. A., Milliken, C. S., & Wilk, J. E. (2014). PTSD treatment for soldiers after combat deployment: Low utilization of mental health care and reasons for dropout. *Psychiatric Services, 65*, 997-1004.
2. Sloan, D. M., Sawyer, A. T., Lowmaster, S. E., Wernick, J., & Marx, B. P. (in press). Narrative writing as an exposure treatment for PTSD: Does the evidence support its use? *Journal of Contemporary Psychotherapy.*
3. Neuner, F., Onyut, P.L., Ertl, V., Odenwald, M., ...& Elbert, T. (2008). Treatment of posttraumatic stress disorder by trained lay counselors in an African refugee settlement: a randomized controlled trial. *Journal of Consulting and Clinical Psychology. 76*, 686-94.
4. Hoge, C.W. (2011). Interventions for war-related posttraumatic stress disorder. Editorial. *JAMA, 306*, 549-551.
5. Agency for Healthcare Research and Quality. (2013). *Psychological and Pharmacological Treatments for Adults with Posttraumatic Stress Disorder (PTSD)* (AHRQ Publication No. 92).Retrieved from author's website.
6. Sloan, D. M., & Marx, B. P. (2004). A closer examination of the structured written disclosure procedure. *Journal of Consulting and Clinical Psychology, 72*, 165-175.
7. Sloan, D. M., Marx, B. P., & Epstein, E. M. (2005). Further examination of the exposure model underlying written emotional disclosure. *Journal of Consulting and Clinical Psychology, 73*, 549-554.

8. Sloan, D. M., Marx, B. P., Epstein, E. M., & Lexington, J. (2007). Does altering the instructional set affect written disclosure outcome?. *Behavior Therapy, 38,* 155-168.
9. Sloan, D. M., Marx, B. P., & Greenberg, E. M. (2011). A test of written emotional disclosure as an intervention for posttraumatic stress disorder. *Behaviour Research and Therapy, 49*, 299-304.
10. Hamblen, J. L., Schnurr, P. P., Rosenberg, A., & Eftekhari, A. (2009). A guide to the literature on psychotherapy for PTSD. *Psychiatric Annals, 39*, 348-354.
11. Pennebaker, J. W. & Beall, S. K. (1986) Confronting a traumatic event. Toward an understanding of inhibition and disease. *Journal of Abnormal Psychology, 95,* 274–281.
12. Baker, A. S., Litwack, S. D., Clapp, J. D., Beck, J.G., & Sloan, D. M. (2014). The Driving Behavior Survey as a measure of behavioral stress responses to MVA-related PTSD. *Behavior Therapy, 45*, 444-453.
13. Imel, Z. E., Laska, K., Jakupcak, M., & Simpson, T. L. (2013). Meta-analysis of dropout in treatments for posttraumatic stress disorder. *Journal of Consulting and Clinical Psychology, 81*, 394-404.
14. Schnurr, P. P., Friedman, M. J., Foy, D. W., Shea, M. T., Hsieh, F. Y., Lavori, P. W., ... & Bernardy, N. C. (2003). Randomized trial of trauma-focused group therapy for posttraumatic stress disorder: Results from a Department of Veterans Affairs cooperative study. *Archives of General Psychiatry, 60*, 481-489.
15. Monson, C. M., Gradus, J. L., Young-Xu, Y., Schnurr, P. P., Price, J. L., & Schumm, J. A. (2008). Change in posttraumatic stress disorder symptoms: Do clinicians and patients agree? *Psychological Assessment, 20*, 131-138.
16. Greene, C. J., Morland, L. A., Durkalski, V. L., & Frueh, B. C. (2008). Noninferiority and equivalence designs: issues and implications for mental health research. *Journal of Traumatic Stress, 21*, 433-439.
17. Cohen J. (1988) Statistical power for the behavioral sciences, 2nd Ed. Lawrence Erlbaum, Hillsdale, NJ.
18. Hintze, J. (2004) NCSS and PASS. Number Cruncher Statistical Systems. Kaysville, Utah. Website: www.ncss.com.
19. Piaggio, G., Elbourne, D. R., Pocock, S. J., Evans, S. J., Altman, D. G., & CONSORT Group. (2012). Reporting of noninferiority and equivalence randomized trials: extension of the CONSORT 2010 statement. *JAMA*, *308*, 2594-2604.
20. Piaggio, G., Elbourne, D. R., Altman, D. G., Pocock, S. J., & Evans, S. J. (2006). Reporting of noninferority and equivalence randomized trials: An extension of the CONSORT statement. *Journal of the American Medical Association, 295*, 1152-1160.
21. Weathers, F.W., Blake, D.D., Schnurr, P.P., Kaloupek, D.G., Marx, B.P., & Keane, T.M. (2013). *The Life Events Checklist for DSM-5 (LEC-5)* [Measurement Instrument]. Retrieved from the National Center for PTSD website.
22. Weathers, F.W., Blake, D.D., Schnurr, P.P., Kaloupek, D.G., Marx, B.P., & Keane, T.M. (2013). *The Clinician-Administered PTSD Scale for DSM-5 (CAPS-5)* [Measurement Instrument]. Retrieved from the National Center for PTSD website.
23. Weathers, F.W., Litz, B.T., Keane, T.M., Palmieri, P.A., Marx, B.P., & Schnurr, P.P. (2013) *The PTSD Checklist for DSM-5 (PCL-5)* [Measurement Instrument]. Retrieved from the National Center for PTSD website.
24. American Psychiatric Association. (1994). *Diagnostic and statistical manual of mental disorders: DSM- IV (4th ed).* Washington, DC: Author.
25. Schnurr, P.P., Friedman, M., Weathers, F.W., & Marx, B.P. (2013). *Assessing PTSD according to the DSM-5.* Pre-meeting institute presented during the annual meeting of the International Society for Traumatic Stress Studies (ISTSS).
26. Sheehan, D. V., Lecrubier, Y., Sheehan, K. H., Amorim, P., Janavas, J., Weiller, E., et al. (1998). The Mini-International Neuropsychiatric Interview (MINI): The development and validation of a structured diagnostic psychiatric interview for DSM-IV and ICD-10. *The Journal of Clinical Psychiatry, 59*, 22–33.
27. Vogt, D. S., Smith, B. N., King, L. A., King, D. W., Knight, J. A., & Vasterling, J. J. (2013). Deployment Risk and Resilience Inventory-2 (DRRI-2): An updated tool for assessing psychosocial risk and resilience factors among service members and veterans. *Journal of Traumatic Stress, 26*, 710-717.
28. Kroenke, K., Spitzer, R. L., & Williams, J. B. W. (2001). The PHQ-9: Validity of a brief depression severity measure. *Journal of General Internal Medicine, 16,* 606-613.
29. Cameron, I. M., Crawford, J. R., Lawton, K., & Reid, I. C. (2008). Psychometric comparison of PHQ-9 and HADS for measuring depression severity in primary care. *British Journal of General Practice, 58,* 32-36.
30. Spitzer, R. L., Kroenke, K., Williams, J. B., & Lowe, B. (2006). A brief measure for assessing generalized anxiety disorder: The GAD-7. *Archives of Internal Medicine, 166,* 1092-1097.
31. Lowe, B., Decker, O., Muller, S., Brahler, E., Schellberg, D., Herzog, W., & Herzberg, P. Y. (2008). Validation and standardization of the Generalized Anxiety Disorder Screener (GAD-7) in the general population. *Medical Care, 46*, 266-274.
32. Kroenke, K., Spitzer, R. L., Williams, J. B., & Lowe, B. (2010). The Patient Health Questionnaire Somatic, Anxiety, and Depressive Symptom Scales: A systematic review. *General Hospital Psychiatry, 32,* 345-359.

33. Babor, T. F., Higgins-Biddle, J. C., Saunders, J. B. & Monteiro, M. G. (2001). *The Alcohol Use Disorders Identification Test: Guidelines for Use in Primary Care, 2nd Edition.* Geneva, Switzerland: World Health Organization.
34. Saunders, J. B., Aasland, O. G., Babor, T. F., De La Fuente, J. R. & Grant, M. (1993). Development of the Alcohol Use Disorders Identification Test (AUDIT): WHO collaborative project on early detection of persons with harmful alcohol consumption-II. *Addiction, 88,* 791-804.
35. Reinert, D. F., & Allen, J. P. (2007). The Alcohol Use Disorders Identification Test: An update of research findings. *Alcoholism: Clinical and Experimental Research, 31,* 185-199.
36. Sobell, L. C., Agrawal, S., Sobell, M. B., Leo, G. I., Young, L. J., Cunningham, J. A., Simco, E. R. (2003). Comparison of a Quick Drinking Screen with the Timeline Followback for individuals with alcohol problems. *Journal of Studies on Alcohol, 64,* 858-861.
37. Roy, M., Dum, M., Sobell, L. C., Sobell, M. B., Simco, E. R., Manor, H., & Palmerio, R. (2008). Comparison of the Quick Drinking Screen and the alcohol Timeline Followback with outpatient alcohol abusers. *Substance Use and Misuse, 43,* 2116-2123.
38. Heatherton, T. F., Kozlowski, L. T., Frecker, R. C., & Fagerstrom, K. O. (1991). The Fagerstrom Test for Nicotine Dependence: A revision of the Fagerstrom Tolerance Questionnaire. *British Journal of Addiction, 86,* 1119-1127.
39. Ebbert, J. O., Patten, C. A., & Schroeder, D. R. (2006). The Fagerstrom Test for Nicotine Dependence – Smokess Tobacco (FTND-ST). Addictive Behaviors,31, 1716-1721.
40. Ferketich, A. K., Wee, A. G., Schultz, J., & Wewers, M. E. (2007). A measure of nicotine dependence for smokeless tobacco users. *Addictive Behaviors, 9,* 1970-1975.
41. Morin, C.M. (1993). *Insomnia: Psychological Assessment and Management.* New York, NY: The Guilford Press.
42. Bastien, C. H., Vallieres, A., Morin, C. M., (2001). Validation of the Insomnia Severity Index as an outcome measure for insomnia research. *Sleep Medicine, 2*(4), 297-307.
43. Chung, F., Yegneswaran, B., Liao, P., Chung, S. A., Vairavanathan, S., Islam, S., Khajehdehi, A., & Shapiro, C. M. (2008). STOP questionnaire: A tool to screen patients for obstructive sleep apnea. *Anesthesiology, 108,* 812-921.
44. Yu, L., Buysse, D. J., & Germain, A. (2012). Development of short forms from the PROMIS Sleep Disturbance and Sleep-Related Impairment item banks. *Behavioral Sleep Medicine, 10,* 6-24.
45. Buysse, D. J., Yu, L., Moul, D. E., Germain, A., Stover, A., Dodds, N. E., Johnston, K. L., Shablesky-Cade, M. A., & Pilkonis, P. A. (2010). Development and validation of patient-reported outcome measures for sleep disturbance and sleep-related impairments. *Sleep, 33,* 781-792.
46. Schwab, K. A., Baker, G., Ivins, B., Sluss-Tiller, M., Lux, W., & Warden, D. (2006). The Brief Traumatic Brain Injury Screen (BTBIS): Investigating the validity of a self-report instrument for detecting traumatic brain injury (TBI) in troops returning from deployment in Afghanistan and Iraq. [Abstract]. *Neurology, 66*(5)(Supp. 2), A235.
47. Schwab, K. A., Ivins, B., Cramer, G., Johnson, W., Sluss-Tiller, M., Kiley, K., Lux, W. & Warden, D. (2006). Screening for traumatic brain injury in troops returning from deployment in Afghanistan and Iraq: Initial investigation of the usefulness of a short screening tool for traumatic brain injury. *Journal of Head Trauma Rehabilitation, 22*(6), 377-389.
48. Metalsky, G. I., & Joiner, T. E. (1997). The Hopelessness Depression Symptom Questionnaire. *Cognitive Therapy and Research, 21,* 359-384.
49. Batterham, P. J., Ftanou, M., Pirkis, J., Mackinnon, A. J., Beautrais, A., Fairweather-Schmidt, A. K., & Christensen, H. (2014, December 15). A systematic review and evaluation of measures for suicidal ideation and behaviors in population-based research. *Psychological Assessment.* Advance online publication. http://dx.doi.org/10.1037/pas0000053
50. Nock, M. K., Holmberg, E. B., Photos, V. I., & Michel, B. D. (2007). Self-Injurious Thoughts and Behaviors Interview: Development, reliability, and validity in an adolescent sample. *Psychological Assessment, 19,* 309-317.
51. Kazis, L. E., Selim, A., Rogers, W., Ren, X. S., Lee, A., & Miller, D. R. Veterans RAND 12-Item Health Survey (VR-12): A White Paper Summary. Unpublished manuscript. http://www.hosonline.org/surveys/hos/download/veterans_rand_12_item_health_survey_white_paper_summary.pdf
52. Marx, B. P. (2013). *Development and validation of a PTSD-related impairment scale.* Retrieved from www.dtic.mil/cgi-bin/GetTRDoc?AD=ADA585414
53. Larsen, D. L., Attkisson, C. C., Hargreaves, W. A., & Nguyen, T. D. (1979). Assessment of client/patient satisfaction: development of a general scale. *Evaluation and Program Planning, 2*(3), 197-207.
54. Tracey, T.J., & Kokotovic, A.M. (1989). Factor structure of the Working Alliance Inventory. *Psychological Assessment, 1*, 207–210.
55. Munder, T., Wilmers, F., Leonhart, R., Linster, H. W., & Barth, J. (2010). Working Alliance Inventory-Short Revised (WAI-SR): Psychometric properties in outpatients and inpatients. *Clinical Psychology & Psychotherapy, 17*, 231-239.
56. Devilly, G. J., & Borkovec, T. D. (2000). Psychometric properties of the credibility/expectancy questionnaire. *Journal of Behavior Therapy and Experimental Psychiatry, 31*, 73-86.

57. Borkovec, T. D & Nau, S. D. (1972). Credibility of analogue therapy rationales. *Journal of Behaviour Therapy and Experimental Psychiatry, 3*, 257-260.
58. P.A. Resick, personal communication, February 22, 2010
59. E.A. Hembree, personal communication, February 23, 2010
60. E. B. Foa, personal communication, February 25, 2010

12.1 Measurement Bibliography:

13.0 TIME REQUIRED TO COMPLETE THE RESEARCH (including data analysis). Approximately 4 years from the time participant recruitment starts.

14.0 STUDY CLOSURE PROCEDURES At the completion of the study a protocol closure report will be submitted for review. At the time of study closure, all links between PHI and the study data will be destroyed unless the participant has also agreed to participation in the STRONG STAR Repository approved by the UTHSCSA IRB (HSC20100475H). Informed consent documents will be kept for 3 years past the closure of the study IAW 32CFR219 and the HIPAA authorizations will be kept for 6 years past the closure of the study IAW 45 CFR160-164 before being destroyed.

15.0 Funding:

Funding Agency: Defense Health Program, Defense Medical Research and Development Program, Department of Defense Psychological Health and Traumatic Brain Injury Research Program, Investigational Treatments for TBI and PTSD Clinical Trial Award Congressionally Directed Medical Research Program Grant ID: PT140164, PI: Denise Sloan

16.0 Description of Assessments:

1. **Demographics and Military Service Characteristics Form**. The Demographics and Military Service Characteristics Form measures standard demographics (race, gender, age) and military service information (e.g., rank).

2. **Life Events Checklist for DSM-5** (LEC-5**;** 21). The LEC-5 includes the same list of 16 different potentially traumatic life events from the original LEC that are commonly associated with PTSD symptoms and designed to facilitate PTSD diagnosis (21). There is also a blank for specifying an additional stressful event not encompassed in the 16 events. For the CAPS-5, the LEC-5 will be used in identifying the index event and focus of PTSD treatment. For each potentially traumatic life event, respondents rate their experience of that event on a 6-point nominal scale (1 = happened to me, 2 = witnessed it, 3 = learned about it, 4 = part of my job, 5 = not sure, and 6 = doesn't apply). The primary addition to the LEC-5 is a category involving occupational exposure ("for example, paramedic, police, military, or other first responder"). There has not been a publication on the psychometric properties of the LEC-5, but the measure is nearly identical to the original LEC. In a group of 108 undergraduate psychology students the LEC demonstrated good convergence with the Traumatic Life Events Questionnaire (average kappa = .55) and correlated with the Posttraumatic Stress Disorder CheckList – Civilian version (reliability coefficients .34 to .48). The LEC demonstrated good test-retest reliability over 7 days (all kappa statistics except one for "caused serious injury / death of another" > .52). In 131 combat veterans the LEC was related in the predicted directions with other measures of psychopathology known to be associated with potentially traumatic life events as assessed with the Posttraumatic Stress Disorder CheckList – Military version, Clinician-Administered PTSD Scale, and the Mississippi Scale for Combat-Related PTSD. The LEC-5 be administered at baseline and at 10, 20, and 30-week follow-up assessments.

3. **Deployment Risk and Resiliency Inventory-2** (DRRI-2; 27) Combat Experiences subscale and Postbattle Experiences subscales. The DRRI-2 is a suite of 17 individual scales that assess key deployment-related risk and resilience factors with demonstrated implications for veterans' long-term health. The Combat Experiences and Postbattle Experiences subscales will be administered at baseline to assess stressful deployment experiences. The DRRI-2 Combat Experiences subscale and Postbattle Experiences subscales will only be administered at baseline.

4. **Clinician Administered PTSD Scale for DSM-5** (CAPS-5; 22). The Clinician-Administered PTSD Scale (CAPS-5) is a structured diagnostic interview and gold standard for assessing PTSD. The scale also assesses social and occupational functioning, dissociation, and the validity of symptom reports. The CAPS was revised to

accommodate the changes made in DSM-5, to reduce administration time, and to facilitate learning administration and scoring procedures. The CAPS-5 now uses only a single 5-point ordinal rating scale to measure symptom severity. Symptom severity ratings combine information about symptom frequency and intensity obtained by the interviewer. At the same time, the CAPS-5 was revised with an eye towards maintaining backwards compatibility with the DSM-IV version of the instrument. Because the measure is new, psychometrics and diagnostic cutoffs are still being evaluated and there are no formal scoring rules yet. The CAPS-5 will be administered at baseline and at 10, 20, and 30-week follow-up assessments.

5. **PTSD Checklist for DSM-5** (PCL-5; 23)**.** The PTSD Checklist for DSM-5 is similar in form to the PTSD Checklist (PCL) based on the DSM-IV (24). The PCL-5 is a 20-item self-report measure, selected for its dimensional sensitivity. Scoring is based on how much the patient has been bothered by the symptoms in the past month on a scale from "0 = not at all" to "4 = extremely." Although extensive empirical testing has not yet been conducted on this measure, initial results suggest that the PCL-5 has psychometric properties commensurate to its predecessor (α = .96 for the total scale; α = .84 test-retest reliability; 25). The PCL will be included in the proposed study to monitor PTSD symptom severity during the treatment phase. The PCL-5 will be administered at baseline, at each treatment session, and at 10, 20, and 30-week follow-up assessments.

6. **Insomnia Severity Index** (ISI; 41). The ISI is a 7-item self-report measure that assesses perceived severity of insomnia. Each item uses a 5-point Likert type scale from 0 to 4, with higher numbers corresponding to greater sleep problems. The items sum to produce a total score (range 0 – 28). The ISI has an internal consistency alpha coefficient of 0.74, and has shown convergent validity with other measures such as the Pittsburgh Sleep Quality Index (r = 0.67), the Dysfunctional Beliefs and Attitudes about Sleep (r = 0.55), and sleep diaries (r ranges from 0.32-0.91; 42). This measure will be administered at baseline and at each follow-up.

7. **Snoring, Tired, Observed, Blood Pressure** (STOP; 43) Sleep Apnea Screen. To better understand sleep disturbance associated with PTSD and PTSD treatment, the STOP screen will be administered to screen for sleep apnea. The STOP is a four-item questionnaire developed and validated in 211 pre-operative surgical patients. Based on the endorsement of 2 or more questions, the sensitivity of the STOP ranged from 66% to 80% as compared with the apnea-hypopnea index (AHI) of polysomnography depending upon the AHI cut-off used. Individuals answering "yes" to 2 or more of the questions will be advised that they may be at risk for having sleep apnea and advised that they may want to speak with their primary care provider to consider referral for an overnight sleep evaluation. This measure will be administered at baseline.

8. **Patient Reported Outcomes Measurement Information System** (PROMIS) Sleep Disturbance and Sleep-Related Impairment short forms (44). The PROMIS Sleep Disturbance and Sleep-Related Impairment short forms are self-report measures of past-week sleep disturbance and past-week sleep-related impairment, respectively, derived from the larger PROMIS item banks (45). Each short-form measure includes 8 items, with most items (symptoms) scored in intensity from 1 ("not at all") to 5 "(very much"). Each measure has shown strong reliability and construct validity (44). This measure will be administered at baseline and at each follow-up.

9. **History of Head Injuries** (modified Defense and Veterans Brain Injury Center [DVBIC] 3-Item Screening Tool). We will use a modified version of the Defense and Veterans Brain Injury Center (DVBIC) 3-Item Screening Tool (46, 47) that was used in STRONG STAR. This instrument, initially called the Brief Traumatic Brain Injury Screen (BTBIS), was used as the gold standard for the diagnosis of TBI in a sample of soldiers returning from duty in Iraq and/or Afghanistan (47). As recommended by the DVBIC, the 3-Question Screen will be considered positive when the participant endorses an injury (question 1) and altered consciousness (question 2, items A-E) for the worst head injury sustained while deployed. The form was modified for STRONG STAR and now CAP to capture the number of injuries, and to answer question 2 based on the worst injury; the original form does not recognize the possibility of multiple head injuries during deployment. As the 3-Question Screen does not query head injuries prior to deployment, an additional four questions have been added to solicit information about each head injury sustained outside of deployment. This measure will be administered at baseline and at each follow-up.

10. **Veterans RAND 12-Item Short Form Health Survey** (VR-12). The VR-12 is a 12-item health questionnaire that was developed from, and explains 90% of the reliable variance of, the longer VR-36 (51). Its items are sampled from each of the eight health domains from the VR-36: physical functioning, role limitations due to physical problems, bodily pain, general health perceptions, energy/vitality, social functioning, role limitations due to

emotional problems, and mental health. Also, there are two summary scales: a physical component summary (PCS) and a mental component summary (MCS). Each item includes a 5-point response scale ranging from "no, none of the time" to "yes, all of the time." The VR-36 has been widely used, distributed and documented in the Veterans Health Administration. Higher scores indicate better health. This measure will be administered at baseline and at each follow-up.

11. **Fagerstrom Test for Nicotine Dependence** (FTND; 38). The Fagerstrom is a 6-item self-report measure that assesses severity of nicotine dependence. Questions probe both quantity of nicotine use (e.g., number of cigarettes per day) and pattern of use (e.g., time to first cigarette in morning). Respondents choose among response options, each of which is assigned a numerical value, with higher numbers corresponding to greater nicotine dependence. Scores on all items are summed to create a severity index with a range of 0 to 10, with higher scores indicating more severe dependence. The Fagerstrom scale has been shown to have high convergent validity with biochemical indices of nicotine use, and the measure has shown acceptable internal consistency (38). A review of 26 studies of the psychometric characteristics of the Fagerstrom found that it is a reliable instrument for measuring nicotine dependence in diverse settings and populations (39). This measure will be administered at baseline and at each follow-up.

12. **Fagerstrom Test for Nicotine Dependence – Smokeless Tobacco** (FTND-ST). This is a modified version of the Fagerstrom Test that focuses on smokeless tobacco use, whereas the original Fagerstrom focuses exclusively on smoking. Like the FTND, the FTND-ST is a 6-item self-report measure of severity of nicotine dependence that has demonstrated convergent validity with biochemical indices of nicotine use (39, 40). As on the original FTND, respondents choose among response options, each of which is assigned a numerical value, with higher numbers corresponding to greater nicotine dependence. Scores on all items are summed to create a severity index (range = 0–10). This measure will be administered at baseline and at each follow-up.

13. **Health Questionnaire** - The Health Questionnaire measures medical and mental health diagnoses that respondents have received, medical board and disability status, medications taken, and caffeine use. The version of the Health Questionnaires used at follow-ups also probes emergency room use, hospitalizations, mental health treatments, military status changes, and any important new life events or changes since the time of the last assessment.

14. **Mini International Neuropsychiatric Interview** (MINI; 26) is a widely used structured psychiatric diagnostic instrument. Responses to the interviewer's questions are rated as either "yes" or "no." This brief interview (~15 minutes) was validated against the much longer Structured Clinical Interview for DSM-IV. For the proposed study, the psychotic module of the MINI will be used at baseline to assessment at baseline assessment to determine study eligibility. The MINI psychotic module will only be administered at baseline.

15. **Patient Health Questionnaire-9** (PHQ-9; 28). The PHQ-9 is a widely used and well-validated instrument for measuring the severity of depressive symptoms. It consists of 9 items that assess both affective and somatic symptoms related to depression and depressive disorders; these 9 items correspond to the diagnostic criteria for DSM MDD. Respondents rate the frequency with which they have been bothered by depressive symptoms within the past two weeks on a scale ranging from 0 ("not at all") to 3 ("nearly every day"). Scores on all items are summed to obtain a total severity score. Scores reflect no significant depressive symptoms (0-4), mild depressive symptoms (5-9), moderate depressive symptoms (10-14), moderately severe depressive symptoms (15-19), and severe depressive symptoms (>19). Respondents also indicate the degree to which their depressive symptoms have made it difficult for them to do their work, take care of things at home, or get along with other people, from "not difficult at all" to "extremely difficult." The PHQ-9 has high internal consistency (e.g., alpha ranging from .83 to .92; 29), and correlates strongly with other measures of depression (28). The PHQ-9 will be administered at baseline, at each treatment session, and at 8, 20, and 30-week follow-up assessments.

16. **Depressive Symptom Index – Suicidality Subscale** (DSI-SS; 48). The DSI-SS will be used to assess current suicidal ideation. The DSI-SS is a 4-item self-report measure of suicidal ideation that focuses on ideation, plans, perceived control over ideation, and impulses for suicide. It is being used as a core measure in the Military Suicide Research Consortium. Scores on each item range from 0 to 3, with higher scores reflecting greater severity of suicidal ideation. Instructions will instruct the participants to respond based on the past two weeks. A systematic review of measures of suicidal ideation and behaviors found that the DSI-SS had evidence of excellent

internal consistency and concurrent validity (49). This measure will be administered at baseline and at each follow-up.

17. **Self-Injurious Thoughts and Behaviors Interview short form** (SITBI; 50). The SITBI is a structured interview assessing the historical presence, frequency, and characteristics of self-injurious and suicidal thoughts and behaviors. The short form version of the SITBI, with 72 items total if no skip-outs are used (i.e., the patient endorses the initial item in each module), will be administered at baseline by an Independent Evaluator, who will instruct the participants to answer the questions based on their entire lifetime of experience. The SITBI has shown high interrater reliability, test-retest reliability, and concurrent validity (50). This measure will be administered at baseline and at each follow-up.

18. **Generalized Anxiety Disorder Screener** (GAD-7; 30). The GAD-7 will be used to assess generalized anxiety symptomology. This is a 7-item measure that asks participants to rate the frequency with which they have been bothered by anxiety symptoms within the past two weeks on a scale ranging from 0 (“not at all”) to 3 (“nearly every day”). Scores on all items are summed to obtain a total severity score. Scores reflect no significant anxiety symptoms (0-4), mild anxiety symptoms (5-9), moderate anxiety symptoms (10-14), and severe anxiety symptoms (>15). Respondents also indicate the degree to which their anxious symptoms have made it difficult for them to do their work, take care of things at home, or get along with other people, from “not difficult at all” to “extremely difficult.” The GAD-7 has been shown to have high internal consistency (e.g., $\alpha = .89$; 31) and has been shown to reliably discriminate between anxious and non-anxious diagnostic groups (32). This measure will be administered at baseline and at each follow-up.

19. **Alcohol Use Disorders Identification Test** (AUDIT; 33) self-report version. The AUDIT will be used to identify people with hazardous or harmful patterns of alcohol consumption and to index the severity of these problems. It will be administered as a self-report form. The AUDIT is a 10-item screening measure, developed by the World Health Organization (WHO), with three subscales (alcohol consumption, drinking behavior, and alcohol-related problems) that are scored on a 4-point scale for a highest possible total score of 40. The AUDIT has good internal consistency (αs = .80-.93) as well as sensitivity and specificity (34; see 35 for review). The AUDIT’s time-frame is the last 12 months. Therefore, for trials without long-term follow-up, the AUDIT will be administered only at baseline. The AUDIT will only be administered at baseline.

20. **Quick Drinking Screen** (QDS; 36) self-report version. The QDS will be used to measure alcohol consumption. It consists of 4 items probing frequency and quantity of alcohol consumption. It will be administered in a self-report form. The QDS has been validated against the Timeline Followback daily estimation measure of alcohol use, and it shows good psychometric properties (36, 37). The QDS’s time-frame will be modified to match the “last two weeks.” This measure will be administered at baseline and at each follow-up.

21. **Brief Inventory of Psychosocial Functioning** (B-IPF; 52). This is a 7-item self-report instrument measuring respondents’ level of functioning in seven life domains: romantic relationship, relationship with children, family relationships, friendships and socializing, work, training and education, and activities of daily living. Respondents indicate the degree to which they had trouble in the last 30 days in each area on a 7-point scale ranging from “0 = Not at all” to “6 = Very much.” The B-IPF has demonstrated concurrent validity, and the full 80-item IPF from which it was created has strong test-retest reliability and internal consistency (52). This measure will be administered at baseline and at each follow-up.

22. **Credibility and Expectancy Questionnaire (CEQ)**. The CEQ is a 6-item measure that was designed to assess treatment expectancy and rationale credibility for use in clinical outcomes studies (56). It has been expanded from a 5-item measure designed primarily to assess credibility (57), 4-items of which have been used by both Foa and Resick (58-60), with the name Expectancy of Therapeutic Outcomes (ETO). The 6-item CEQ assesses both whether the person cognitively understands how the therapy works (credibility) as well as whether the person affectively believes that the therapy will work for them personally (expectancy). The 6-item CEQ has been tested in 217 individuals including 68 male Vietnam veterans and 58 female spouses, 69 individuals diagnosed with general anxiety disorder who had received treatment, and 22 individuals who had received either Cognitive Based Therapy (CBT) or Eye Movement Desensitization and Reprocessing (EMDR) for the treatment of PTSD. The scale demonstrated high internal consistency (alpha coefficients ranged from 0.84 to 0.85). Test-retest reliability over a one-week period was found to be 0.82 for expectancy and 0.75 for credibility. The CEQ was able to differentiate between two treatment rationales in one study, one with and one without an encompassing theory,

while maintaining equivalence between three rationales in another study. Responses to four questions are scored using a 9-point Likert scale (1= not at all, 9= extremely). Responses to two of the questions are scored using an 11-point Likert Scale (0% to 100%). The combined responses are used to generate a score for credibility and another score for expectancy. This measure pre- therapy CEQ will be administered at baseline and the post-therapy CEQ will be administered at end of treatment.

23. **The Client Satisfaction Questionnaire** (CSQ; 53) is an 8 item measure of participant satisfaction with treatment. This measure will be administered at the last session of treatment in order to examine whether participants are satisfied with the treatment they have received.

24. **The Working Alliance Inventory, Short Form** (WAI-SF, 54) is a measure to index the degree of therapeutic cohesion between the client and the therapist. The WAI-SF demonstrates good internal consistency and convergent validity (54,55). Participants will complete the WAI-SF at the end of treatment.

Appendices:
Appendix A: WET manual
Appendix B: CPT-C manual
Appendix C: Study Flyers
Appendix D: Pre-Screen Phone Script
