# Supplementary material for: Effect of Written Exposure Therapy vs Cognitive Processing Therapy on Increasing Treatment Efficiency Among Military Service Members With Posttraumatic Stress Disorder: A Randomized Noninferiority Trial
Source: JAMA Netw Open. 2022 Jan 11;5(1):e2140911. doi: 10.1001/jamanetworkopen.2021.40911 (PMC8753496; doi:10.1001/jamanetworkopen.2021.40911)
Supplement: Supplement 2. — eMethods 1. CAPS-5 Protocol eMethods 2. Therapists [file jamanetwopen-e2140911-s002.pdf]

Supplemental Online Content

Sloan DM, Marx BP, Resick PA, et al; STRONG STAR Consortium. Effect of written exposure therapy vs cognitive processing therapy on increasing treatment efficiency among military service members with posttraumatic stress disorder: a randomized noninferiority trial. *JAMA Netw Open*. 2022;5(1):e2140911. doi:10.1001/jamanetworkopen.2021.40911

eMethods 1. CAPS-5 Protocol

eMethods 2. Therapists

This supplemental material has been provided by the authors to give readers additional information about their work.

© 2022 Sloan DM et al. *JAMA Network Open.*

eMethods 1. CAPS-5 Protocol

Across the STRONG STAR affiliated studies, the CAPS-5 was administered by a pool of masters- or doctoral-level staff that received standardized training to a criterion of reliability and subsequent certification as independent evaluators (IEs). IEs were blind to treatment condition and treatment status at the beginning and end of treatment. Approximately 3% of all CAPS-5 interviews across the CAP were randomly selected for review on twice monthly calibration calls to establish interrater reliability and to prevent rater drift. We generated a kappa coefficient based on comparisons of decisions about the presence or absence of PTSD by an IE with an expert evaluator co-rating the audio-taped interviews. We also generated a correlation coefficient based on the total CAPS-5 severity scores by the IE and the expert evaluator. The inter-rater reliability of CAPS-5 case decisions (N = 148; *Cohen's kappa* = .87, $p < .01$) and the correlation of severity scores between raters were both excellent ($r = .96$, $p < .01$).

© 2022 Sloan DM et al. *JAMA Network Open.*

eMethods 2. Therapists

A total of nine doctoral- and masters-level therapists completed a 2-day CPT workshop and 4 hour WET workshop prior to treating participants in the study. The same therapists were used for both arms of the study. All therapists received weekly supervision from the first or the fifth author, who listened to recorded therapy sessions. Independent CPT and WET clinicians rated 15% of randomly selected recorded treatment sessions using adherence and competence rating forms selected from prior studies (Resick et al., 2015; Resick et al., 2016).

Raters completed assessments of "unique and essential elements" for each rated session. Raters assessed adherence and competence according to a 7-point scale (1 = poor, 7 = excellent, with satisfactory as the midpoint) for WET and a 5-point scale (1= poor, 5 = excellent) for CPT-C. Average adherence by WET therapists was 6.04 (SD = 0.68), and average adherence by CPT therapists was 3.92 (SD = 0.72). The average therapist competence score for WET was 6.41 (SD = 0.76), and the average CPT therapist competence score was 4.15 (SD = 0.86). Across all rated sessions in both conditions, 94.3% of adherence ratings were "good" to "excellent," and none were below "satisfactory."

© 2022 Sloan DM et al. *JAMA Network Open.*
