# Supplementary material for: Effect of Written Exposure Therapy vs Cognitive Processing Therapy on Increasing Treatment Efficiency Among Military Service Members With Posttraumatic Stress Disorder: A Randomized Noninferiority Trial
Source: JAMA Netw Open. 2022 Jan 11;5(1):e2140911. doi: 10.1001/jamanetworkopen.2021.40911 (PMC8753496; doi:10.1001/jamanetworkopen.2021.40911)
Supplement: Supplement 3. — Data Sharing Statement [file jamanetwopen-e2140911-s003.pdf]

Data Sharing Statement

Sloan. Effect of Written Exposure Therapy vs Cognitive Processing Therapy on Increasing Treatment Efficiency Among Military Service Members With Posttraumatic Stress Disorder. *JAMA Netw Open*. Published January 11, 2022. doi:10.1001/jamanetworkopen.2021.40911

Data
Data available: Yes
Data types: Deidentified participant data
How to access data: Requests for data should be sent to petersona3@uthscsa.edu
When available: beginning date: 12-01-2022

Supporting Documents
Document types: None

Additional Information
Who can access the data: researchers whose proposed used of the data has been approved.
Types of analyses: for any purpose that has been approved.
Mechanisms of data availability: with a signed data access agreement
